# Humanized dual-targeting antibody–drug conjugates specific to MET and RON receptors as a pharmaceutical strategy for the treatment of cancers exhibiting phenotypic heterogeneity

**DOI:** 10.1038/s41401-024-01458-7

**Published:** 2025-01-21

**Authors:** Minghai Wang, Qi Ma, Sreedhar Reddy Suthe, Rachel E. Hudson, Jing-ying Pan, Constantinos Mikelis, Miao-jin Zhu, Zhi-gang Wu, Dan-rong Shi, Hang-ping Yao

**Affiliations:** 1 State Key Laboratory for Diagnosis and Treatment of Infectious Diseases, National Clinical Research Center for Infectious diseases, First Affiliated Hospital, Zhejiang University School of Medicine, Hangzhou, 310003 China; 2 Department of Pharmaceutical Sciences, Jerry H. Hodge School of Pharmacy, Texas Tech University Health Sciences Center, Amarillo, 79106 TX USA; 3 Cancer Biology Research Center, Jerry H. Hodge School of Pharmacy, Texas Tech University Health Sciences Center, Amarillo, 79106 TX USA; 4 Comprehensive Genitourinary Cancer Center, First Affiliated Hospital of Ningbo University, Ningbo, 315000 China; 5 Translational Research Laboratory for Urology, The Key Laboratory of Ningbo City, Ningbo, 315000 China

**Keywords:** cancer heterogeneity, receptor tyrosine kinases, dual-targeting antibody-drug conjugates, pharmacokinetic, toxic activity, xenograft tumor model

## Abstract

Cancer heterogeneity, characterized by diverse populations of tumorigenic cells, involves the occurrence of differential phenotypes with variable expressions of receptor tyrosine kinases. Aberrant expressions of mesenchymal–epithelial transition (MET) and recepteur d’origine nantais (RON) receptors contribute to the phenotypic heterogeneity of cancer cells, which poses a major therapeutic challenge. This study aims to develop a dual-targeting antibody–drug conjugate (ADC) that can act against both MET and RON for treating cancers with high phenotypic heterogeneity. Through immunohistochemical staining, we show that MET and RON expressions are highly heterogeneous with differential combinations in more than 40% of pancreatic and triple-negative breast cancer cases. This expressional heterogeneity provides the rationale to target both receptors for cancer therapy. A humanized bispecific monoclonal antibody specific to both MET and RON (PCMbs–MR) is generated through IgG recombination using monoclonal antibody sequences specific to MET and RON, respectively. Monomethyl auristatin E is conjugated to PCMbs–MR to generate a dual-targeting ADC (PCMdt–MMAE), with a drug-to-antibody ratio of 4:1. Various cancer cell lines were used to determine PCMdt-MMAE-mediated biological activities. The efficacy of PCMdt–MMAE in vivo is evaluated using multiple xenograft tumor models. PCMdt–MMAE shows a favorable pharmacokinetic profile, with a maximum tolerated dose of ~30 mg/kg in mice. Toxicological studies using Sprague–Dawley rats reveal that PCMdt–MMAE is relatively safe with slight-to-moderate, temporary, and reversible adverse events. Functionally, PCMdt-MMAE induces a robust internalization of both MET and RON and causes a large-scale cell death in cancer cell lines exhibiting MET and RON heterogeneous co-expressions. Both in vitro and in vivo studies demonstrate that the dual-targeting approach in the form of an ADC is highly effective with a long-lasting effect against tumors exhibiting MET/RON heterogeneous phenotypes. Hence, we can suggest that a dual-targeting ADC specific to both MET and RON can be employed as a novel therapeutic strategy for tumors with expressional phenotypic heterogeneity.

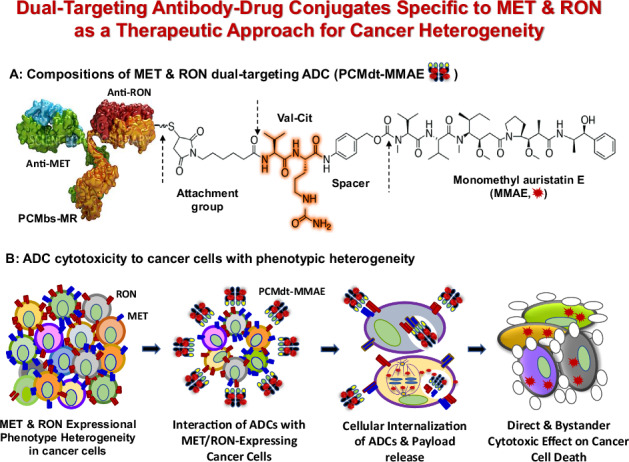

## Introduction

Cancer heterogeneity is the presence of diverse cell populations with varying molecular and cellular phenotypes within a tumor mass [1–3]. This concept reflects genetic aberrations among individual cancer cells, which can lead to differential gene expression patterns, altered proliferation potentials, and intrinsic survival capabilities [1–3]. A phenotypic manifestation of cancer heterogeneity is the aberration of receptor tyrosine kinases (RTKs), such as that of human epidermal growth factor receptors 1 and 2 (HER1 and HER2, respectively) [4, 5]. Cancer heterogeneity presents a significant clinical challenge to oncological therapy. Hence, it becomes necessary to develop innovative strategies and novel therapeutics exhibiting higher efficacy and which can also handle complications arising from cancer heterogeneity [1–4].

The antibody–drug conjugate (ADC), a biotherapeutic, is a novel category of medication that is being increasingly employed for the targeted delivery of chemotherapy drugs to solid tumors. An ADC has three constituents: a monoclonal antibody (mAb), a linker, and a payload [6]. Various biotherapeutics, including ADCs that target cell-surface proteins such as HER2 and TROP-2, are used to address cancer heterogeneity [5–7]. Trastuzumab emtansine (T-DM1)—an ADC—is employed to treat cancer heterogeneity [5–7]. However, the success of T-DM1 is limited to breast tumors expressing high levels of HER2 [5–7]. Recently, development of ADCs using payloads with relatively low potency and a higher drug-to-antibody ratio (DAR), such as trastuzumab deruxtecan (DS-8201a), has been approved for cancers exhibiting phenotypic heterogeneity [8, 9]. DS-8201a is composed of a novel linker–payload system comprising a humanized anti-HER2 antibody, an enzymatically cleavable peptide-linker, and DXd—a novel topoisomerase I inhibitor. Anti-HER2 antibodies localize ADCs to HER2-expressing tumor cells and release DXd, thereby inducing the breakage of double-stranded DNA and apoptosis of HER2-positive tumor cells. As a payload, DXd also has the advantage of a short half-life, which can lead to a bystander effect. Even though DXd has a DAR of 8, it exhibits favorable pharmacokinetics (PK). Therefore, it is effective not only against tumors with a high HER2 expression, but also against those with low levels of RTKs [8–10].

Mesenchymal-epithelial transition (MET) and recepteur d’origine nantais (RON) belong to a unique subfamily of RTKs with similar structures and functions [11, 12]. At cellular levels, aberrant expression of MET and RON activates a biological program that facilitates cancer cell invasive growth, distant metastasis, and chemoresistance [13, 14]. These activities are channeled through various intracellular signaling pathways, such as mitogen-activated protein kinase and phosphatidylinositol 3-kinase pathways [11–14], which are essential for tumor initiation, progression, malignancy, and stemness. Pathologically, aberrant MET and/or RON expression can lead to characteristic heterogeneous appearances of cancer cells, including those of the colon, breast, and pancreas cancers [15–22]. Additionally, a strong signaling crosstalk between MET and RON is noted. This crosstalk acts as a regulatory feedback loop that enhances the tumorigenic phenotype of cancer cells. It may also act as a signal compensation pathway that supports the growth of cancer cells and provides them with a survival advantage allowing them to evade targeted therapies [12]. These abnormalities make MET and RON valuable phenotypic markers that give rise to cancer heterogeneity [11–14].

Clinical interventions using MET and/or RON-targeted therapeutics are attracting intense research attention [11, 12, 23, 24]. Small molecule kinase inhibitors, such as cabozantinib and crizotinib, have been approved for oncological application [23, 24]. Various therapeutic mAbs specific to MET or RON have also been developed [25, 26]. Recently, ADCs targeting MET and/or RON have been reported [27–38]. Several ADCs targeting MET, such as ABBV-399 [28, 29], TR1801-ADC [27], and SHR-A1403 [30], are currently being investigated through various clinical trials (www.clinicaltrials.gov). These ADCs show considerable clinical and preclinical antitumor activities, as well as acceptable tolerability and safety profiles, in several solid tumors, including non-small cell lung cancer, pancreatic cancer, gastric cancer, and colon cancer [39]. ADC telisotuzumab vedotin, formerly called ABBV-399, has been granted the Breakthrough Therapy Designation Status by the FDA (www.fda.gov). In addition, ADCs targeting RON, such as Zt/g4-monomethyl auristatin E (Zt/g4-MMAE) and PCM5B14-duocarmycin (PCM5B14-DCM), have been preclinically validated [32–38]. Hence, it is clear that both anti-MET and anti-RON ADCs are effective in inhibiting and/or eradicating xenograft tumors with favorable PK profiles and manageable toxicological activities in primates, including humans [27–38]. These findings highlight the potential of using anti-MET and anti-RON ADCs as a novel strategy for cancer therapy. However, tumor-acquired resistance and heterogeneous target expression make the application of single-target ADC therapies highly challenging. Resolving these issues is crucial to advancing effective ADC-based cancer therapies [40].

This study investigates the development and validation of a novel dual-targeting ADC specific to both MET and RON for the treatment of cancers exhibiting phenotypic heterogeneity. The highly heterogenetic tumorigenic expression of MET and RON in cancer cells prompted us to use a dual-targeting ADC as a therapeutic approach to eliminate cancer cells displaying heterogeneous phenotypes. The objective of the study is to determine whether the dual-targeting ADC is therapeutically effective against xenograft tumors co-overexpressing both MET and RON. In particular, it aims to confirm the therapeutic activity of this dual-targeting ADC on the growth inhibition of tumors with heterogeneous MET and RON expressions.

## Materials and methods

### Primary tumor samples and immunohistochemical staining

Primary samples of pancreatic ductal adenocarcinoma (PDAC, 244 cases) and triple negative breast cancer (TNBC, 236 cases) were obtained from the Department of Pathology at the First Affiliated Hospital, Zhejiang University School of Medicine (Hangzhou, China). Immunohistochemical (IHC) staining was performed for MET and RON using rabbit anti-MET IgG antibodies (Abcam, Waltham, MA) and PCM-7F11, a mouse anti-RON mAb (PCM Targetech, Dallas, TX), respectively. The MET and RON expression levels were determined using a previously described semi-quantitative method [17].

### Cell lines and reagents

The following established cell lines obtained from the American Type Cell Culture (Manassas, VA) were employed: breast cancer: T-47D, TNBC HCC1806, HCC1937, HCC2185, MDA-MB-231, MDA-MB468, and SUM52PE; colorectal cancer: HCT116, HT29, PDAC ASPC-1, BxPC-3, and FG; gastric cancer: Hs746T; and lung cancer: H358. In addition, we employed the following cell lines as well: monkey bronchial 4MBr-5, Martin–Darby canine kidney (MDCK), mouse endothelial MS-1, and fibroblast NIH3T3 cell lines. Mouse anti-RON mAbs Zt/g4 and PCM5B14 and rabbit anti-RON polyclonal IgG antibody (R#5029) were used [32–34]. Mouse mAb PCM-MET01 specific to one of the MET extracellular sequences (generated by the PCM Targetech LLC, Dallas, Texas, USA) was employed [41]. The specificity and sensitivity of PCM-MET01 to MET were confirmed by immunofluorescence (IF) analysis and enzyme-linked immunosorbent assay (ELISA). Goat anti-mouse or human IgG antibodies labeled with fluorescein isothiocyanate (FITC) were acquired from Jackson ImmunoResearch (West Grove, PA, USA).

### Generation and characterization of bispecific mAb PCMbs-MR

The bispecific mAb specific to both MET and RON (PCMbs-MR) was obtained from PCM Targetech LLC (Dallas, TX, USA). Anti-MET PCM-MET01 and anti-RON PCM5B14 were used as the starting materials for constructing PCMbs-MR. Individual sequences from complementarity-determining regions of anti-MET mAb PCM-MET01 and anti-RON mAb PCM5B14 were grafted into human IgG1/κ acceptor frameworks using knobs-into-holes and immunoglobulin domain crossover technologies [42, 43] to generate humanized MET and RON bispecific recombinant IgG molecules (PCMbs-MR and IgG1/κ, respectively) [32, 33]. We obtained ~40 mg of PCMbs-MR. The specificity and sensitivity of PCMbs-MR were determined through IF, ELISA, and Western blot analyses.

### Generation of PCMbs-MR-based dual-targeting ADCs

MMAE was conjugated to PCMbs-MR to generate PCMdt-MMAE and achieve a DAR of 4:1 [32, 33]. Maleimidocaproyl–valine–citrulline–*p*-aminobenzoyl–oxycarbonyl–MMAE was obtained from Levena BioPharma (San Diego, CA, USA). PCMbs-MR was conjugated with maytansinoid derivative 1 (DM1) to form dual-targeting ADC PCMdt-DM1 [34]. PCM-MET01 was conjugated with MMAE to produce PCM-MET01-MMAE. Anti-RON ADC Zt/g4-MMAE was used [32]. A control ADC was generated by conjugating regular human IgG with MMAE to produce RhIgG-MMAE, as described above. Average DARs were calculated from the integrated areas of the DAR species through hydrophobic interaction chromatography (HIC) [32–34].

### Analyses of MET and RON expressions

Cell-surface RON and MET intensities were analyzed through flow cytometry [32]. Anti-MET mAb PCM-MET01 and anti-RON mAb PCM5B14 were included for comparison. Briefly, 1 × 10^6^ cells per sample were first incubated with 2 µg/mL of PCMbs-MR or other antibodies and then with rabbit anti-human or mouse IgG coupled with FITC. Regular human IgG was used as the control. Immunofluorescence intensities from individual samples were analyzed by flow cytometry. To quantitatively measure the total amount of cell-surface RON and MET, we conducted an immunofluorescence assay using QIFIKIT^®^ reagents (DAKO, Carpentaria, CA, USA) [32–34]. Briefly, cells (1 × 10^6^ cells/mL in PBS) were treated with 5 μg/mL of anti-MET mAb or anti-RON mAb, followed by parallel incubation using QIFIKIT^®^ beads and goat F(ab′)2 F0479. Once a calibration curve was obtained, the number of RON receptors on the cell surface was determined through interpolation.

### Assays for PCMbs–MR-induced MET and RON internalization

PCMbs–MR-induced MET and RON internalization was performed based on the methods described previously [32–34]. Briefly, IF intensities from control cells treated with a mixture of PCM-MET01 and PCM5B14 at 4 °C were set as 100%. Cells from the experimental groups were treated at 37 °C with 5 µg/mL of PCMbs–MR for various time periods. Immunofluorescence was analyzed by flow cytometry along with FITC-coupled anti-mouse IgG. The time required to achieve 50% reduction in cell-surface MET and RON, known as internalization efficacy (IE_50_), was calculated for individual cell lines using a previously described method [32–34].

### Assays for cell cycle, cell viability, and cell death

These assays were performed using previously described methods [32–34]. Changes in the cell cycle were analyzed after labeling the cells with propidium iodide using an Accuri Flow Cytometer [32]. The MTS assay was conducted to determine the cell viability after 96 h of PCMdt–MMAE treatment [32]. The number of dead cells was determined by performing the trypan blue exclusion assay [32]. All experiments were performed three times to validate their reproducibility and accuracy.

### Pharmacokinetic profiles and toxic activities of PCMdt–MMAE in mice and rats

Female Balb/c mice (three mice per group), with or without BxPC-3 cell-mediated xenograft tumors, were used. A single dose of PCMdt-MMAE (3 or 10 mg/kg of body weight) was administered through the tail vein. The amount of MMAE conjugated to PCMbs–MR in plasma was determined by using a MMAE ADC ELISA kit (Eagle Biosciences Inc., Nashua, NH, USA; www.eaglebio.com/product/mmae-adc-elisa-kit) [32–34]. The PK parameters were calculated using the Phoenix WinNonlin^TM^ software package (Certara, Princeton, NJ, USA) [32–34]. The maximum tolerated dose of PCMdt-MMAE in Balb/c mice (three mice per dose) was determined through a single tail-vein injection of 10, 30, and 60 mg/kg of PCMdt-MMAE. Changes in mouse activity, behavior, bodyweight, and survival for up to 12 days were documented. Acute toxicities of PCMdt–MMAE in male Sprague–Dawley rats (three animals per group) were studied following a single injection of 10 or 30 mg/kg of PCMdt–MMAE. Post-injection clinical signs, including changes in animal activity, responsiveness, bodyweight, and survival, were monitored for up to 28 days. Blood samples were collected at different time points for hematological and blood chemistry measurements. Tissue samples from individual animals were harvested at the end of the study for histological examination.

### Mouse xenograft tumor model and PCMdt–MMAE treatment

All animal experiments were approved by the institutional animal care committee. Female athymic nude mice (6-week-old) (Taconic, Cranbury, NJ, USA) were subcutaneously injected with 5 × 10^6^ of the following cancer cell lines [32–34]: HCC1806, HT29, FG, T-47D, HCT116, BxPC-3, and H358. The mice were randomized into different groups (five animals per group). Treatment was started when the individual tumors reached the mean tumor volume of ~150 mm^3^. Tumors initiated by HT29^(MET3+/RON3+)^ colon cancer cells were used to compare the efficacy of PCMdt-MMAE, with anti-MET ADC PCM-MET01-MMAE and anti-RON ADC PCM5B14-MMAE, which were employed as references. The mice were administered a single dose of 10 mg/kg of PCMdt–MMAE, PCM–MET01–MMAE, or PCM5B14–MMAE via their tail veins. Then, T-47D^(MET2+/RON3+)^ and FG^(MET3+/RON2+)^ cell lines were utilized to perform dose-dependent studies. PCMdt–MMAE was administered at doses of 1, 3, 7, 10, and 15 mg/kg in a Q12×2 schedule. The objective was to determine the duration of PCMdt–MMAE-mediated activity. The H358^(MET2+/RON3+)^, HCT116^(MET2+/RON3+)^, and BxPC-3^(MET3+/RON2+)^ cells were used. A single-injection regimen of PCMdt–MMAE comprised a dose of 10 mg/kg. Mice treated with RhIgG-MMAE served as the control. The tumor volumes were measured every four days [34]. All animals were euthanized when their tumor volumes exceeded 2500 mm^3^ or if the tumors ulcerated through the skin. At the end of the study, tumors from the individual animals were collected, weighed, and histologically analyzed.

### Statistical analysis

GraphPad Prism 6 software was used for statistical analysis. Results are shown as mean ± SD. The data between the control and experimental groups were compared using Student’s *t*-test. Statistical differences at *P* < 0.05 were considered significant.

## Results

### Expression profiles of MET and RON in primary PDAC and TNBC samples

**Fig. 1:**
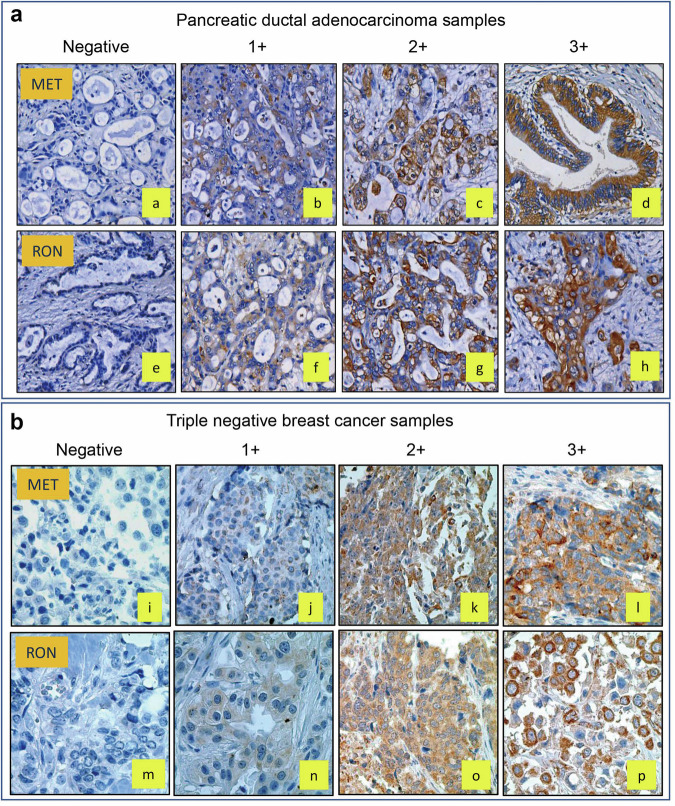
Immunohistochemical staining and analysis of MET and RON expression in primary PDAC and TNBC samples. **a** Differential expression of MET and RON in 244 primary PDAC samples. **b** Differential expression of MET and RON in 236 TNBC samples. IHC staining of MET and RON using their corresponding specific antibodies, respectively, was performed as detailed in Materials and Methods. MET and RON expression **lev**e**ls** were determined using semi-quantitative methods [14]. Staining intensities of MET and RON were determined as negative, weak, moderate, or strongly positive. The area reactivity of staining was set as 0 = < 1%, 1 = 2%–30%, 2 = 31%–70%, and 3 = 71%–100%. The combined scores that define MET and RON expressions were as follows: 0, negative; 1–2, low; 3–4, moderate; and 5–6, high, corresponding to the scale of 0, 1^+^, 2^+^, and 3^+^, respectively. Various patterns of MET or RON expression with different IHC staining intensities were observed. The representative images are presented. IHC immunohistochemistry, MET mesenchymal-epithelial transition, RON recepteur d’Origine nantais, PDAC pancreatic ductal adenocarcinoma, TNBC triple negative breast cancer.

A summary of the pathological features of 244 PDAC and 236 TNBC samples is given in Supplementary Table 1. The IHC staining of MET and RON was performed in the paired tumor tissue using specific antibodies. The immunoreactive intensity, patterns, and positivity were evaluated by referencing the FDA-approved criteria for EGFR positivity [44]. Representative images of MET and RON were obtained using the negative to positive staining technique with different intensities (Fig. 1a, b). The individual PDAC and TNBC samples exhibited similar patterns of MET and RON expressions, but with heterogeneous appearances. Both samples exhibited predominantly membrane, predominantly cytoplasmic, and mixed staining patterns (Fig. 1a, b), suggesting differential expression patterns of MET and RON in a single tumor mass. Hence, we can suggest that MET and RON were differentially expressed in the PDAC and TNBC samples with different staining intensities and patterns, reflecting the heterogeneous nature of individual cancer cells.

A summary of MET and RON expression in 244 PDAC samples is given in Table 1. A total of 218 and 225 PDAC samples expressed MET (89.35%) and RON (92.21%), respectively. Tumors from 117 PDCA patients showed low-to-moderate levels of MET expression (53.67%). Similarly, 136 cases had low-to-moderate RON expression levels (60.44%). Overexpression was observed in 101 cases for MET (46.33%) and 89 cases for RON (39.56%). Among the 218 cases showing MET positivity, 157 cases exhibited co-expression with RON (72.02%). Similarly, in the 225 cases demonstrating RON positivity, 141 exhibited co-expressed RON (62.67%). Matched co-overexpression was observed only in 23.39%–22.67% of the total number of cases for both MET and RON.

**Table 1:** Immunohistochemical analysis of MET and RON expression by immunohistochemical staining in primary pancreatic ductal adenocarcinoma and triple negative breast cancer samples^a^.

Cancer samples stained with anti-MET or RON mAbs	Expression profiles of Met and RON in 244 primary pancreatic ductal adenocarcinoma samples				Expression profiles of Met and RON in 236 primary triple negative breast cancer samples			
	MET expression		RON expression		MET expression		RON expression	
	cases	(%)	cases	(%)	cases	(%)	Cases	(%)
Negative	26	10.65	19	7.79	38	16.10	51	21.61
Positive	218	89.35	225	92.21	198	83.90	185	78.39
Low to moderate	117	53.67	136	60.44	108	54.54	94	50.81
overexpression	101	46.33	89	39.56	90	45.46	91	49.19
Matched coexpression	157	72.02	141	62.67	127	64.14	134	72.43
Matched overexpression	51	23.39	51	22.67	60	30.30	60	32.42

Pathological digit evaluation was performed as previously described [38]. Levels of the IHC staining were determined as negative (0), weak positive (1), moderate positive (2), or strong positive (3). Two pathologists blinded to clinical information independently scored MET and RON positivity in each sample.

^a^Immunohistochemical (IHC) staining was performed as detailed in Materials and Methods. Levels of MET and RON expression was determined in a semi-quantitative method in reference to the FDA-approved criterion for EGFR expression in tumor samples [43].

Among the 236 TNBC samples, 198 samples expressed MET (83.90%) and 185 samples expressed RON (78.37%) (Table 1). Tumors from 108 patients showed low-to-moderate levels of MET expression (55.45%). Similarly, 94 cases had low-to-moderate levels of RON expression (50.81%). Overexpression was observed in 90 cases for MET (45.46%) and 91 cases for RON (49.19%). Among the total 198 MET-positive cases, 127 co-expressed RON (64.14%), while among the 185 RON-positive cases, 134 exhibited MET expression. The matched co-overexpression in both cases was 30.30% and 32.42%, respectively (Table 1). These findings indicate that MET and RON are co-expressed in variable combinations at high frequencies, suggesting that MET and RON expressional heterogeneity is a pathogenic feature in both pancreatic and breast cancers.

### Generation of MET and RON bispecific mAbs and dual targeting ADCs

**Fig. 2:**
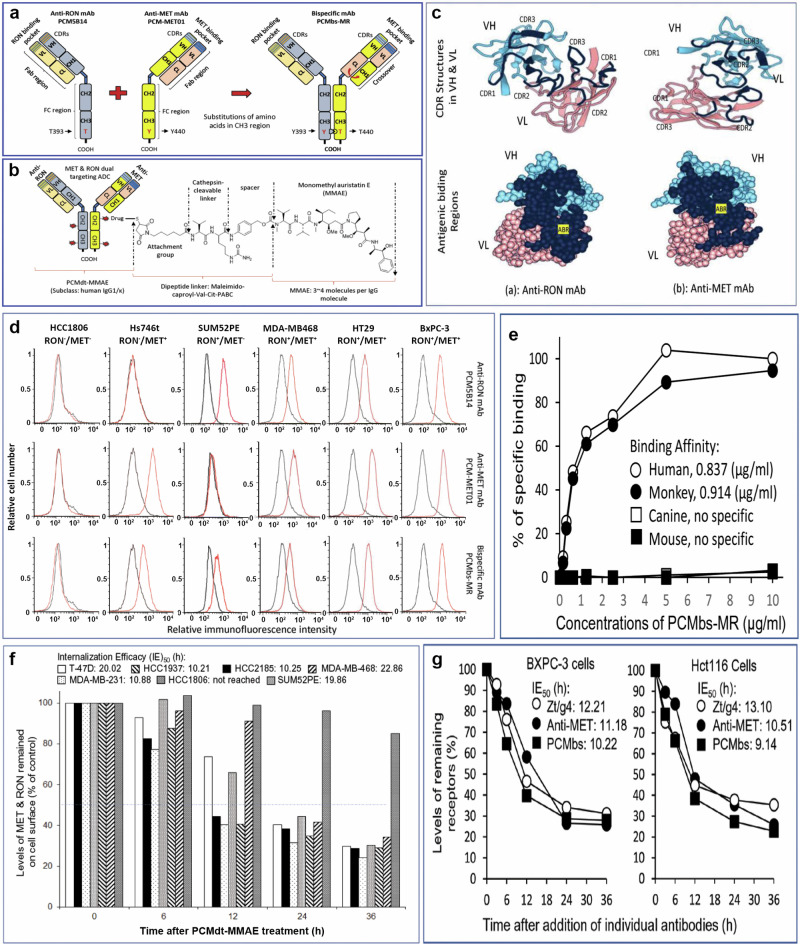
Generation and characterization of bispecific monoclonal antibody PCMbs-MR. **a** Schematic structure of PCMbs-MR generated through cDNA recombination from anti-MET mAb PCM-MET01 and anti-RON mAb PCM5B14. A single heavy chain and light chain, representing both anti-MET mAb and anti-RON mAb, respectively, is presented. The knob-into-hole was created in the CH3 domains of both anti-MET and anti-RON mAbs [42]. The crossover was conducted between the CH1 domain of the heavy chain and the CL domain of the light chain from the anti-MET mAb accordingly [43]. **b** PCMbs-MR is conjugated with MMAE through a cleavable dipeptide linker to form the dual-targeting ADC PCMdt-MMAE [32–34]. The drug-to-antibody ratio (DAR) was calculated as 4.13:1. **c** Structural 3D analysis of individual complementary-determining regions (black) grafted into the variable regions of humanized heavy chain and light chain (top panel). Antigen-binding surfaces in the variable regions of the humanized anti-MET and anti-RON mAbs are depicted as dark blue (bottom panel). **d** Cell surface immunofluorescent analysis of PCMbs-MR binding to MET and RON. A panel of cancer cell lines expressing variable levels of MET, RON, or both receptors were used. Anti-MET mAb PCM-MET01 and anti-RON mAb PCM5B14 were included for comparison. Briefly, cells at 1 × 10^6^ cells per sample were incubated with 2 µg/mL of PCMbs-MR or other antibodies (red line), followed by rabbit anti-human or mouse IgG coupled with FITC. Regular human IgG was used as the control (black line). Immunofluorescence intensities from individual samples were analyzed using a flow cytometer [32]. Cellular levels of receptor expressions are indicated as follows: HCC1806^(MET-/RON-)^; Hs746T^(MET3+/RON-)^; SUM52PE^(MET-/RON3+)^; MDA-MB468^(MET1+/RON2+)^; HT29^(MET3+/RON3+)^; BxPC-3^(MET3+/RON2+)^; T-47D^(MET3+/RON3+)^; HCC1937^(MET3+/RON3+)^; HCC2185^(MET3+/RON-)^; and MDA-MB-231^(MET3+/RON2+)^. **e** Species specificity of PCMbs-MR. Human HT29, monkey 4MBr-5, canine MDCK, and mouse MS-1 cell lines, known to express both MET and RON, were used. Incubation of cells with PCMbs-MR and specific immunofluorescence analysis were performed as detailed previously [32]. The binding affinity of PCMbs-MR was calculated using the GraphPad Prism 6 software. **f** PCMbs-MR induced cell-surface MET and RON internalization by individual cancer cells. Seven cancer cell lines expressing variable levels of MET, RON, or both receptors were used. PCMbs-MR at 5 µg/mL was used for incubation. Procedures used to determine cell surface receptor internalization were performed as previously described [32–34]. Internalization efficacy (IE_50_) among cell lines was calculated using a previously described method [32–34]. **g** PCMbs-MR-induced internalization efficacy in comparison with anti-MET or anti-RON mAbs. BxPC-3 and HCT116 cell lines expressing high levels of both MET and RON were used. Treatment of cells with mAbs and the methods used to determine IE_50_ were carried out to obtain the individual IE_50_ values [32]. ADC antibody–drug conjugate, FITC fluorescein isothiocyanate, mAb monoclonal antibody, MET mesenchymal-epithelial transition, MMAE monomethyl auristatin E, PCMbs–MR humanized bispecific monoclonal antibody specific to both MET and RON, RON recepteur d’Origine nantais.

Anti-MET mAb PCM-MET01 and anti-RON mAb PCM-5B14 were selected to construct PCMbs-MR—the humanized IgG1 bispecific antibody (Fig. 2a, c). PCMbs-MR was appropriately modified and optimized to ensure the proper formation of bispecific IgG molecules. The resulting PCMbs-MR was then utilized to generate MET and RON dual-targeting ADC PCMdt–MMAE with a DAR of 4:1 (Fig. 2b). PCMbs–MR was specifically validated to interact with cell-surface MET only, RON only, and both MET and RON (Fig. 2d). PCMbs–MR still exhibits considerable binding capacity in tumor cell lines expressing RON or MET alone. We analyzed the species-specificity of PCMbs–MR and found that it binds to MET and RON only from humans and monkeys, not to those from canines or mice (Fig. 2e). It exhibits a combination-binding affinity of 0.836 µg/mL (5.57 nM) for human MET and RON and that of 0.914 µg/mL (6.09 nM) for monkey MET and RON homologs.

The ability of PCMbs-MR to induce cell-surface MET, RON, and both receptor internalization is shown in Fig. 2f. A panel of cell lines with different levels of MET and RON and their combinational expression patterns were used. The average time required for PCMbs–MR to induce a 50% reduction in the MET and RON receptor levels (IE_50_) ranged from 10.21 to 22.86 h. Importantly, PCMbs-MR was found to be effective in inducing the internalization of both MET and RON, as well as their receptors in cell lines exhibiting heterogeneous expression patterns (Fig. 2d–f). A comparison of PCMbs–MR with anti-MET and anti-RON mAbs in receptor internalization using the PDAC cell line BxPC-3^(MET3+/RON2+)^ and colorectal cancer cell line HCT116^(MET2+/RON3+)^, both of which co-express high levels of MET and RON, is shown in Fig. 2g. The average IE_50_ value for PCMbs–MR (9.68 h) is comparable to those of PCM–MET01 (10.85 h) and PCM5B14 (12.66 h). Thus, PCMbs–MR is specific to both MET and RON. As bispecific binding causes a robust internalization of cell-surface MET and RON by cancer cells, PCMbs–MR is an ideal candidate for targeted drug delivery.

### Pharmacokinetic profiles and toxic effects of PCMdt–MMAE

**Fig. 3:**
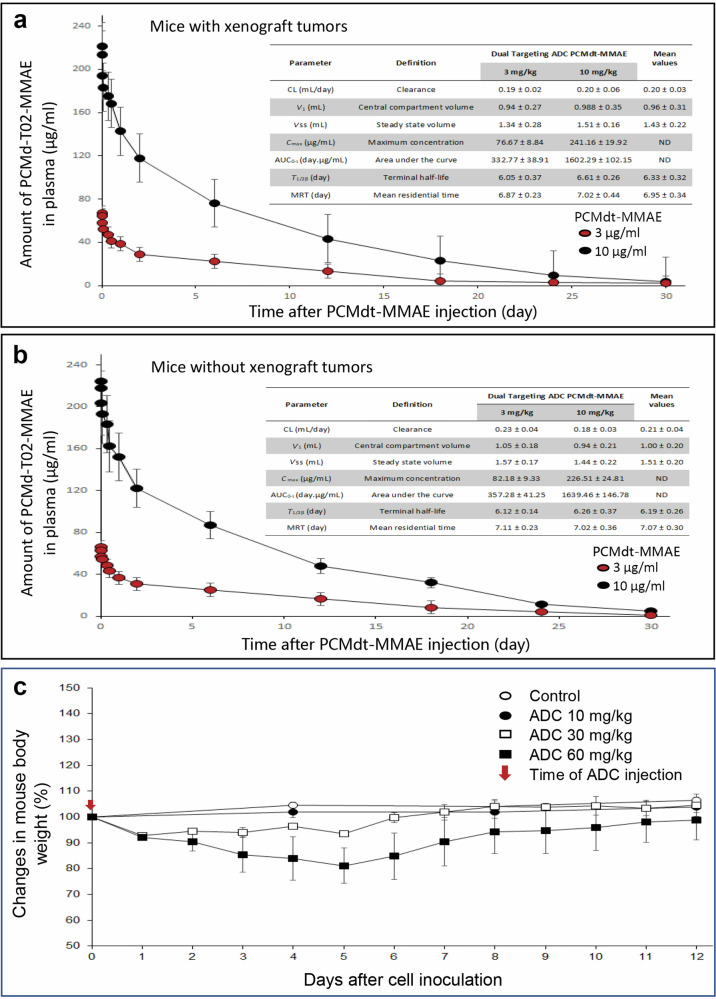
Pharmacokinetic profiles and toxic activities of PCMdt-MMAE in mice. Analysis of PCMdt-MMAE PK profiles: Athymic nude mice (8-week-old) were divided into tumor-bearing (**a**) and tumor-nonbearing (**b**) groups (three animals per group). Mice from the tumor-bearing group were subcutaneously injected with 1 × 10^6^ BxPC-3 cells. When tumor volumes reached ~500 mm^3^, both groups of mice were administered once through the tail vein with 3 or 10 mg/kg of PCMdt-MMAE. Blood samples were collected from individual mice at different time intervals. The amount of MMAE-conjugated PCMbs-MR in plasma was determined by using a MMAE ADC ELISA kit (Eagle Biosciences Inc., Nashua, NH, USA). The PK parameters were calculated using the WinNonlin software package (Certara, Princeton, NJ, USA) [32–34]. **c** Toxic effects of PCMdt-MMAE in vivo: Effect**s** of multiple doses of PCMdt-MMAE on mouse bodyweight were determined by a single administration of PCMdt-MMAE at 10, 30, and 60 mg/kg, respectively. The mice were weighed and monitored for 12 days. The average bodyweight before PCMdt-MMAE injection was 19.8 ± 3.6 g (five mice per group) and set as 100%. ADC, antibody–drug conjugate; MMAE, monomethyl auristatin E; PCMdt-MMAE, monomethyl auristatin E was conjugated to PCMbs–MR to generate the dual-targeting ADC; PCMbs-MR humanized bispecific monoclonal antibody specific to both MET and RON, PK pharmacokinetic

A single-dose injection of 10 or 30 mg/kg of PCMdt–MMAE was injected into mice, with or without BxPC-3 cell-mediated xenograft tumors. The objective was to determine whether the PK profile of PCMdt–MMAE could be altered in mice bearing tumors expressing MET and RON. Mice without tumors were used to determine the target-independent behavior of PCMdt–MMAE. The PK profile of PCMdt–MMAE in the plasma of both tumor-bearing and tumor-nonbearing mice appeared to exhibit the two-compartment model with similar patterns for both low and high doses (Fig. 3a, b). In tumor-bearing mice, PCMdt–MMAE had an average mean plasma clearance of 0.20 mL·d^−1^·kg^−1^, a *t*½ of 6.33 days, and a mean residential period of 6.95 days (Fig. 3a). These values were similar to those in mice without tumors (0.21 mL·d^−1^·kg^−1^, 6.19 days, and 7.07 days, respectively) (Fig. 3b). Overall, data from tumor-bearing mice overlapped with those from tumor-nonbearing mice with 95% prediction intervals. Thus, the presence of tumors with MET and RON expression does not affect the dynamics of PCMdt–MMAE.

To determine the maximum tolerated dose of PCMdt–MMAE, the mice were categorized into three groups based on the single-dose injection of PCMdt–MMAE (10, 30, and 60 mg/kg) (Fig. 3c). Mice injected with 10 mg/kg of PCMdt–MMAE behaved normally during the observation period. The average body weight of the experimental group was comparable to that of control mice, with no significant differences. Slightly distressed activity was observed in mice administered with 30 mg/kg of PCMdt–MMAE. These mice also showed an average reduction of ~5% (*P* > 0.05 in comparison with control mice) in body weight within the first 5 days of PCMdt–MMAE injection. This reduction was, however, recovered on day 6 of the observation period. In contrast, a 20% reduction in body weight was observed in mice treated with 60 mg/kg of PCMdt–MMAE (*P* < 0.05, compared with control mice). The overall bodyweight of these mice remained slightly lower than that of control mice, with a difference of ~3% on day 12. Thus, a single dose of <30 mg/kg of PCMdt–MMAE appears to be relatively safe without any statistical differences in the activity, behavior, or body weight of the animals.

Toxicity profiles were studied using Sprague–Dawley rats with a single-dose injection of PCMdt–MMAE (10 and 30 mg/kg). All animals survived at the end of the study period (28 days). No gross abnormal changes were observed in the daily activity, bodyweight, food consumption, or responsiveness of the animals. However, abnormalities were observed in the hematology and blood workup of the mice (Supplementary Table 2). Notably, 30 mg/kg, not 10 mg/kg, of PCMdt–MMAE caused moderate reductions in leukocytes, as evident by the decrease in the populations of neutrophils, lymphocytes, and monocytes. Blood chemistry analysis revealed varying levels of the increase in blood enzymatic activities, including those of alanine transaminase, alkaline phosphatase, aspartate aminotransferase, and creatine kinase, indicating damage to the liver and other tissues. In addition, moderate myelosuppression in the bone marrow was observed with 30 mg/kg of PCMdt–MMAE. These adverse reactions explain the effects of MMAE [32]. All abnormalities were temporary, reversible, and normalized to baseline levels by the end of the study, suggesting that up to 30 mg/kg of PCMdt–MMAE can be safely used.

### Effects of PCMdt–MMAE on cancer cell cycle, viability, and death

**Fig. 4:**
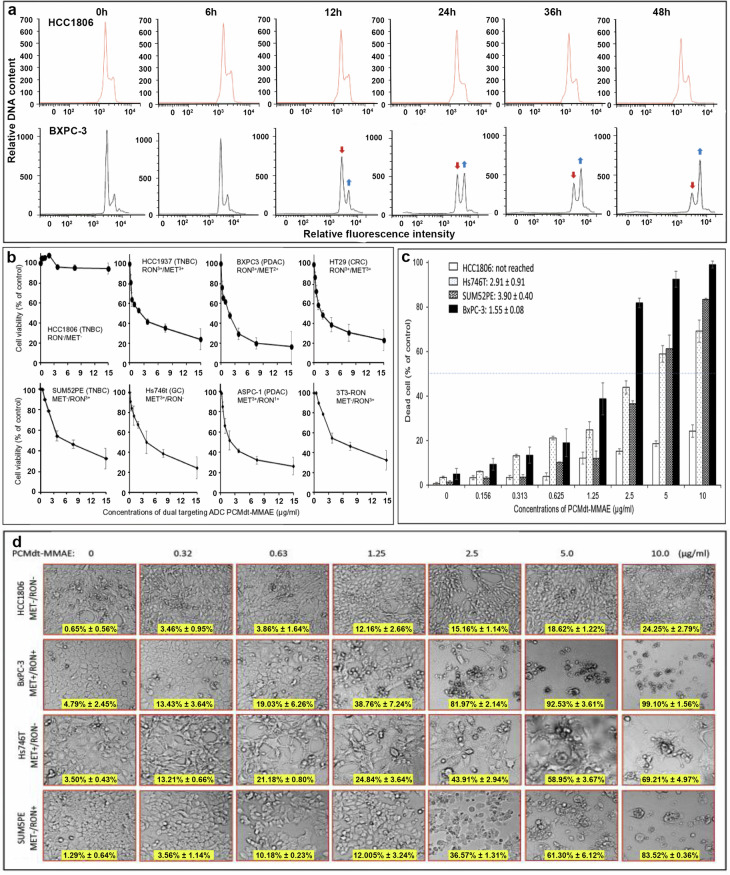
Biological effects of PCMdt-MMAE on CRC cell cycle, survival, and death. **a** Changes in cell cycle: BxPC-3 and control HCC1806 (1 × 10^6^ cells per dish) cells were treated at 37 °C with 5 µg/mL of PCMdt-MMAE for various times. They were then collected, stained with propidium iodide, and analyzed using a flow cytometer [32–34]. Changes in cell cycle were marked with arrows. **b** Reduction of cell viability: A panel of seven cancer cell lines expressing variable levels of MET, RON, or both receptors (5000 or 8000 cells per well in a 96-well plate in triplicate) were treated with different amounts of PCMdt-MMAE for 96 h. HCC1806 cells without MET or RON expression served as the control. Cell viability was determined by the MTS assay [32]. **c** Dose-dependent cell death: Three cancer cell lines expressing MET, RON, or both receptors were treated with different amounts of PCMdt-MMAE for 96 h as described in (**b**). HCC1806 cells were used as the control. At the end of the study, the dead cells were counted using the Trypan blue exclusion assay to determine the percentage of cell death [32]. **d** Morphological evidence of cell death. Treatment of cells with PCMdt-MMAE was performed as described in (**c**). Cellular morphological changes from individual cell lines were observed at 96 h under the Olympus BK-41 inverted microscope and photographed. For all studies described above, the percentages of cell viability and/or cell death and the individual IC_50_ values from individual groups were calculated using the GraphPad Prism 6 software. Results shown here are from one of three experiments with similar results. ADC antibody–drug conjugate, CRC colorectal cancer, MET mesenchymal-epithelial transition, PCMdt-MMAE monomethyl auristatin E was conjugated to PCMbs–MR to generate the dual-targeting ADC, RON recepteur d’Origine nantais

The pathogenic features of cancer cell lines are presented in Supplementary Table 3. The effects of PCMdt–MMAE on the cell cycle were studied using BxPC-3 cells. HCC1806 cells were employed as the control. PCMdt–MMAE treatment of BxPC-3 cells led to dramatic changes in their cell cycles (Fig. 4a). This effect could be observed as early as 12 h after the addition of the ADC and was characterized by a significant reduction in the G_0_/G_1_ phase, a decrease in the S phase, and an increase in the G_2_/M phase. As expected, HCC1806 cells did not respond to PCMdt–MMAE. Quantitative measurements of cell cycle changes are shown in Supplementary Table 4. These results suggest that PCMdt–MMAE has a profound impact on the cell cycle of cancer cells heterogeneously co-expressing MET and RON.

The effect of PCMdt–MMAE on cell viability was studied using six cancer cell lines (HCC1806, BxPC-3, HT29, SUM52PE, Hs746T, and ASPC-1) expressing varying levels of MET and RON, as well as their heterogeneous combinations. PCMdt–MMAE treatment in a dose-dependent manner led to a significant reduction in cell viability (Fig. 4b). The IC_50_ value of PCMdt–MMAE at 96 h was within 1.34–3.78 μg/mL, with an average value of 2.52 μg/mL. A comparison of the ability of PCMdt–MMAE to reduce cancer cell viability with that of anti-MET and anti-RON ADCs is shown in Supplementary Table 5. The average IC_50_ values of PCMdt–MMAE were comparable to those of anti-MET and anti-RON ADCs. Thus, PCMdt–MMAE is effective not only in cancer cells expressing MET, RON, and their heterogeneous combinations at high levels, but also in cells co-expressing both MET and RON at relatively low levels.

We further determined the effect of PCMdt–MMAE on cell death using three cancer cell lines (Hs746T, SUM52PE, and BxPC-3) expressing MET, RON, and their heterogeneous combinations. PCMdt–MMAE can kill cancer cells in a dose-dependent manner, with IC_50_ values of 2.91 µg/mL ± 0.91 for Hs746T^(MET3+/RON-)^, 3.90 µg/mL ± 0.40 for SUM52PE^(MET-/RON3+)^, and 1.55 µg/mL ± 0.08 for BxPC-3^(MET3+/RON2+)^ (Fig. 4c). The morphological observation indicated cell death on a massive scale 96 h after the cells were exposed to PCMdt–MMAE (Fig. 4d), although cellular sensitivities to PCMdt–MMAE-induced cell death varied among the three cell lines tested. Thus, based on the results depicted in Fig. 4c, d, along with those in Fig. 4a, b, it can be suggested that PCMdt–MMAE is active not only in cancer cells with only MET or RON expression, but also in those co-expressing both receptors. The effect of PCMdt–MMAE appears to be manifested through cell cycle arrest, cell viability reduction, and large-scale cell death.

### Therapeutic activity of PCMdt–MMAE against tumors heterogeneously co-expressing MET and RON

**Fig. 5:**
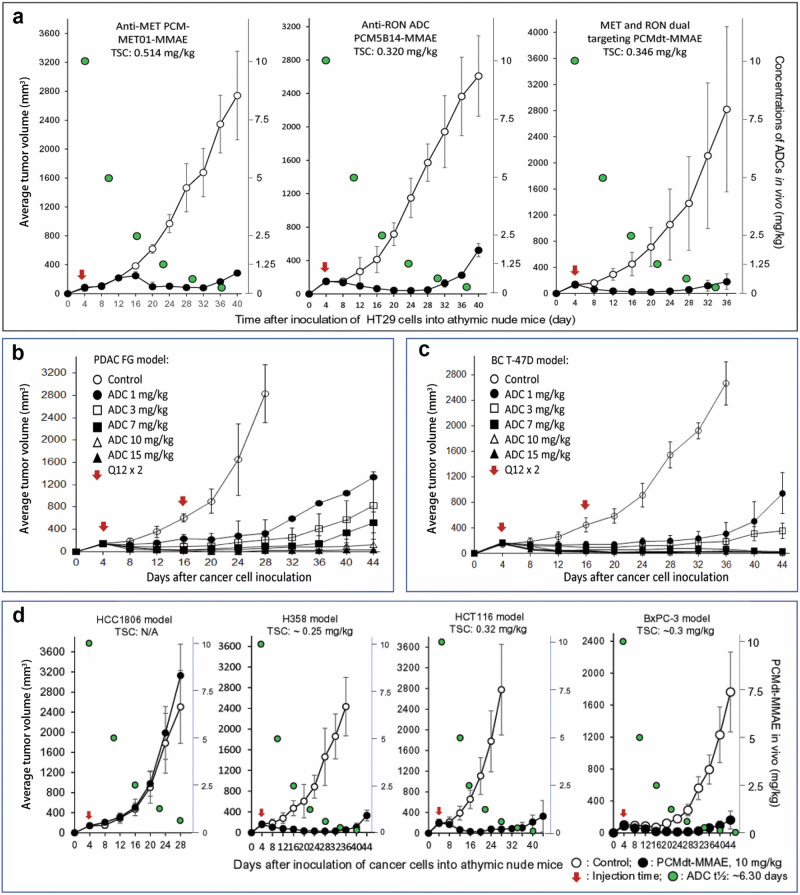
Therapeutic efficacy of PCMdt-MMAE in xenograft tumor models. Mice bearing xenograft tumors were divided into experimental and control groups (five animals per group). ADCs were injected through the tail vein. RhIgG-MMAE was used as the control. Tumor volumes were measured every four days. Individual tumors were collected at the end of the study, photographed, weighed to reach an average value per group, and analyzed for statistical differences using GraphPad Prism 6 software. The tumoristatic concentration (TSC), a minimal dose required to maintain a balance between the growth and inhibition of xenograft tumors, was calculated [32–34]. **a** Therapeutic efficacy of PCMdt-MMAE in comparison with anti-MET or anti-RON ADCs. HT29 cell-mediated xenograft tumors were used. PCMdt-MMAE, PCM-MET01-MMAE, and PCM5B14-MMAE at 10 mg/kg were injected once into mice. Mice treated with RhIgG-MMAE served as the control. Tumor growth was monitored up to day 36 or 40. **b**, **c** Dose-dependent effects of PCMdt-MMAE on xenograft tumor growth. FG and T-47D cell-derived xenograft tumors were used. PCMdt-MMAE from 1, 3, 7, 10, and 15 mg/kg in a Q12×2 schedule was injected once into mice. **d** Duration of PCMdt-MMAE-mediated anticancer activity. Xenograft tumors derived from H358, BxPC-3, and HCT116 cell lines were used. HCC1806 tumors served as the control. PCMdt-MMAE at 10 mg/kg was used for treatment. Tumor growth from each group was monitored until tumor regrowth was visible. ADC antibody–drug conjugate, MET mesenchymal-epithelial transition, PCMdt-MMAE monomethyl auristatin E was conjugated to PCMbs–MR to generate the dual-targeting ADC, RON recepteur d’Origine nantais.

The first objective of our study was to determine the efficacy of PCMdt-MMAE using PCM-MET01-MMAE and PCM5B14-MMAE as references. Tumors initiated by HT29 colon cancer cells^(MET3+/RON3+)^ were used. Individual ADCs were administered in a single dose of 10 mg/kg to tumor-bearing mice. The growth of HT29 cell-mediated tumors was dramatically inhibited among all three groups of mice treated with individual ADCs (Fig. 5a and Table 2A). PCMdt-MMAE exhibited long-lasting antitumor activities up to day 32 without any sign of tumor regrowth. The efficacy of PCMdt–MMAE, as determined by using its tumoristatic concentration (TSC), is comparable to that of anti-MET and anti-RON ADCs without any statistical significance (Fig. 5a).

**Table 2:** A Therapeutic efficacy of PCMdt-MMAE at a single dose of 10 mg/kg in comparison with that of anti-MET ADC PCM-MET-1-MMAE or anti-RON ADC PCM5B14-MMAE against tumor xenografts mediated by colorectal cancer HT29 cells^a^. B Dose-dependent efficacy of PCMdt-MMAE against tumor xenografts mediated by PDAC FG and breast cancer T-47D cells in mouse models^b^. C Therapeutic effect of PCMdt-MMAE on multiple tumor xenografts mediated by T-47D, FG, HT29, and BxPC-3 cell lines in mouse models^c^.

A									
Types of ADCs tested	Therapeutic efficacy of dual targeting ADC in comparison with that of anti-MET or anti-RON ADC								
	Final days of tumor volume measured	Average tumor volume (mm^3^)	Average tumor volume reduction	Final days of tumor collected	Average tumor weight (g)	Average tumor weight reduction	Number of tumors eradicated	% of tumor eradicated	
Anti-MET ADC PCM-MET01-MMAE	Control	40	2,740.94 ± 650.55	0.0	40	1.328 ± 0.52	0.0	0/5	0.0
	Experimental	40	286.01 ± 71.78	**89.57%**	40	0.126 ± 0.11	**90.51%**	0/5	0.0
Anti-RON ADC PCM5B14-MMAE	Control	40	2,608.91 ± 523.51	0.0	40	1.416 ± 0.63	0.0	0/5	0.0
	Experimental	40	532.51 ± 78.45	**79.59%**	40	0.108 ± 0.42	**92.37%**	2/5	40%
Dual Targeting ADC PCMdt-MMAE	Control	36	2,821.03 ± 1,266.81	0.0	36	2.50 ± 2.54	0.0	0/5	0.0
	Experimental	36	179.77 ± 119.30	**93.63%**	36	0.09 ± 0.07	**96.4%**	1/5	20%

B												
Various parameters measured	PCMdt-MMAE in PDAC FG Xenograft tumor model (mg/kg)						PCMdt-MMAE in BC T-47D xenograft tumor model (mg/kg)					
	0	1	3	7	10	15	0	1	3	7	10	15
Days tumor volume measured	28	28	28	28	28	28	36	36	36	36	36	36
Average tumor volume (mm^3^) in each group	2,841.09 ± 468	359.49 ± 178	241.46 ± 103	102.43 ± 35	69.31 ± 11	17.14 ± 6	2,673.66 ± 288	317.55 ± 57	206.42 ± 64	91.42 ± 21	76.91 ± 28	12.30 ± 6
Average tumor volume reduction (%)	0.0	**87.36**	**91.52**	**96.41**	**97.56**	**99.01**	0.0	**88.12**	**92.28**	**96.58**	**97.12**	**99.54**
Days when tumor collected	28	44	44	44	44	44	36	44	44	44	44	44
Average tumor weight (g) in group	2.48 ± 0.42	0.42 ± 0.12	0.27 ± 0.11	0.14 ± 0.23	0.08 ± 0.00	0.02 ± 0.00	1.74 ± 0.39	0.22 ± 0.10	0.20 ± 0.12	0.06 ± 0.00	0.04 ± 0.00	0.02 ± 0.00
Average tumor weight reduction (%)	0.00	**83.06**	**89.11**	**95.35**	**96.77**	**99.19**	0.00	**87.36**	**88.51**	**96.55**	**97.70**	**99.55**
Number of tumors eradicated	0/5	0/5	1/5	3/5	4/5	4/5	0/5	0/5	2/5	4/5	4/5	4/5
Percentages of tumor eradicated (%)	0.0	0.0	20	60	80	80	0.0	0.0	40	80	80	80

C										
Various parameters measured	Therapeutic efficacy of MET and RON dual targeting ADC PCMdt-MMAE on tumor xenografts mediated by multiple cancer cell lines									
	HCC1806 model		H358 model		HCT116 model		HT29 model		BxPC3 model	
	control	ADC	control	ADC	Control	ADC	Control	ADC	Control	ADC
Final days tumor volume measured	28	28	36	40	28	44	36	36	44	44
Average tumor volume (mm^3^) in each group	2504.28 ± 620.50	3129.77 ± 724.36	2435.16 ± 566.71	330.62 ± 103.45	2774.56 ± 875.67	975.76 ± 304.65	2821.03 ± 1266.81	179.77 ± 119.30	1764.40 ± 500.01	160.63 ± 113.27
Average tumor volume reduction (%)	0.0	-24.98	0.0	**86.42**	0.0	**64.83**	0.0	**93.63**	0.0	**90.90**
Days when tumor collected	28	28	36	44	28	44	36	36	44	44
Average tumor weight (g) in group	1.462 ± 0.732	1.694 ± 0.931	1.366 ± 0.574	0.203 ± 0.112	1.572 ± 0.733	0.384 ± 0.206	2.004 ± 2.469	0.072 ± 0.094	0.830 ± 0.503	0.104 ± 0.165
% of average tumor weight reduction	0.0	-15.87	0.0	**85.14**	0.0	**75.57**	0.0	**96.41**	0.0	**87.47**
Number of tumors eradicated	0/5	0/5	0/5	2/5	0/5	1/5	0/5	1/5	0/5	1/5
% of tumor eradicated	0%	0%	0%	**40%**	0%	20%	20%	0%	0%	20%

Bolded data highlight the significant differences among the groups.

^a^MET and RON dual targeting ADC PCMdt-MMAE was injected in a single dose of 10 mg/kg. Anti-MET ADC PCM-MET01-MMAE and anti-RON ADC PCM5B14-MMAE were used for comparison. Tumor growth from individual mice in each group were monitored by measuring tumor volume every four days. At the end of the study, tumors from individual mice were collected, photographed, weighted, and analyzed using GraphPad Prism 6 software. The average reduction in tumor volume from each group and the average reduction in tumor weight from each group were calculated as previously described [32, 34]. The number of tumors eradicated from PCMdt-MMAE treated animals were counted accordingly. Values showing statistical significances of differences (*p* < 0.05) between control and ADC treated animals were bolded.

^b^Tumor growth from individual mice in each group were monitored by measuring tumor volume every four days. At the end of the study, tumors from individual mice were collected, photographed, weighted, and analyzed using GraphPad Prism 6 software. The average reduction in tumor volume from each group and the average reduction in tumor weight from each group were calculated as previously described [32, 34]. The number of tumors eradicated from PCMdt-MMAE treated animals were counted accordingly. Statistical significances (*p* < 0.05) between control and ADC treated animals were bolded in black.

^c^Tumor growth from individual mice in five xenograft tumor models were monitored by measuring tumor volume every four days. At the end of the study, tumors from individual mice were collected, photographed, weighted, and analyzed using GraphPad Prism 6 software. The average reduction in tumor volume from each group and the average reduction in tumor weight from each group were calculated as previously described [32, 34]. The number of tumors eradicated from PCMdt-MMAE treated four groups of animals were counted accordingly. Statistical significances (*p* < 0.05) between control and ADC treated animals were bolded in black.

Our second objective was to determine the dose-dependent activity of PCMdt–MMAE (Fig. 5b, c, and Table 2B). T-47D^(MET2+/RON3+)^ and FG^(MET3+/RON2+)^ cell lines heterogeneously co-expressing MET and RON were utilized. PCMdt–MMAE, which inhibited both FG and T47-D cell-derived tumor growth in a dose-dependent manner, was administered at doses of 1, 3, 7, 10, and 15 mg/kg in a Q12×2 schedule. Notably, tumor growth could be delayed to up to day 28 or 32 with 1 mg/kg of PCMdt–MMAE (Fig. 5b, c, and Table 2B). Long-term inhibition of tumor growth was noted with 7, 10, and 15 mg/kg of PCMdt–MMAE (Fig. 5b, c, and Table 2B). In these cases, no sign of tumor regrowth was observed until day 44. A further analysis of the average tumor number and tumor weight at the end of the study period demonstrated that PCMdt–MMAE reduced tumor weights in a dose-dependent manner (Table 2B). The T-47D tumors exhibited a significant reduction in tumor weight, ranging from 65% to 99%, depending on the PCMdt–MMAE dose. Moreover, PCMdt–MMAE, at all administered doses, was able to eradicate tumors to varying levels (Table 2B). Thus, PCMdt–MMAE is highly effective against tumors heterogeneously co-expressing both MET and RON.

Our final objective was to determine the duration of PCMdt–MMAE-mediated activity. The H358^(MET2+/RON3+)^, HCT116^(MET2+/RON3+)^, and BxPC-3^(MET3+/RON2+)^ cells showing varying levels of MET and RON co-expressions were used. A single-injection regimen of PCMdt–MMAE comprised a dose of 10 mg/kg. The growth of tumors caused by all the aforementioned three cancer cell lines was inhibited upon PCMdt–MMAE administration (Fig. 5d and Table 2C). The average TSC value of PCMdt–MMAE was 0.29 mg/kg for these tumors. Signs of tumor regrowth among the three models tested were observed only around day 36 or 40, equivalent to six *t*½ (~6.3 days) cycles of PCMdt–MMAE. An analysis of the average tumor volume and weight at the end of the study indicated that PCMdt–MMAE is highly effective in inhibiting tumor growth. Average tumor volume reductions of 86.42%, 97.00%, and 90.90% were observed for H358 at day 36, HCT116 at day 28, and BxPC-3 at day 32, respectively. Notably, PCMdt–MMAE exhibited variable tumor-eradicating activities (Table 2C). Thus, a single-dose regimen of 10 mg/kg of PCMdt–MMAE is highly effective with long-lasting activity against tumors initiated by the aforementioned three cancer cell types with varying levels of MET and RON co-expressions.

## Discussion

This study investigated the feasibility of using PCMdt–MMAE for the treatment of cancers exhibiting MET/RON expressional phenotype heterogeneity. IHC staining revealed that MET and RON expressions are highly heterogeneous and exhibit different combinations in primary PDAC and TNBC samples. We have earlier reported 127 cases (67.90%) of breast cancer (TNBC) [45] and 182 cases (80.18%) of pancreatic cancer (PDAC) with RON and MET co-expression [46]. In the present study, we conducted IHC staining of 236 primary PDAC samples and observed MET and RON co-expressions at high frequencies and in varying combinations (Fig. 1 and Table 1). Notably, matched co-expression of these two receptors was noted in more than 60% of samples. Similarly, primary TNBC samples also exhibited a high frequency of MET and RON expression with matched co-expression ranging from 64% to 72%. In PDAC and TNBC samples, the matched MET and RON co-overexpression varied from 22.67% to 32.42%. These findings suggest that MET and RON expressional heterogeneity is a pathogenic feature in both PDAC and TNBC samples. Currently, several ADCs targeting MET are being investigated in various clinical trials for solid tumors that express c-Met. For example, ABBV-399 is in a phase III clinical trial, and TR1801-ADC and SHR-A1403 are in a phase I clinical trial. Based on these results, we can reason that the phenotypic heterogeneity characterized by the co-expression of MET and RON can be utilized to develop a targeted therapeutic intervention. Hence, the use of dual-targeting ADCs can be a promising therapeutic strategy for cancers exhibiting a differential RTK expression phenotype.

We used PCMbs–MR for developing a dual-targeting ADC because of its unique features. First, PCMbs–MR is a recombinant IgG1 specific to both MET and RON. This property is acquired from PCM–MET01 and PCM5B14, both of which are excellent mAbs owing to their good antigen-interacting properties (Fig. 2). Second, PCMbs–MR can rapidly induce the internalization of MET and RON, a process essential for the successful application of dual-targeting ADCs. For instance, PCM5B14 can induce a strong internalization of RON by interacting with the plexin–semaphorin–integrin (PSI) domain in the RON β-chain extracellular sequence [33], which is critical for regulating the RON internalization process [47, 48]. The region recognized by PCM–MET01 in the MET extracellular sequence is currently unknown. However, it is known that PCM–MET01 strongly induces MET internalization with an IE_50_ of ~10 h in a panel of cancer cell lines tested (Fig. 2). This IE_50_ is comparable to that of PCM5B14 (average IE_50_ = 8–12 h). Third, PCMbs–MR is easily conjugated with various payloads using thioether and dipeptide linkage platforms. Although we did not investigate the stability of PCMdt–MMAE under in vivo conditions, we expect that its stability profile is similar to that of anti-RON ADC Zt/g4-MMAE because both of them have similar linker chemistry [32]. This concept has also been confirmed by PK analysis in vivo (Fig. 3a, b). Fourth, the PCMbs–MR-mediated drug delivery was effective in a panel of cancer cell lines tested. More than 70% of cell-surface MET and RON were internalized within 36 h of PCMdt-MMAE treatment. HCC1937 cells expressing ~20,000 RON and ~18,000 MET molecules per cell internalized ~26,600 receptor molecules within 36 h. This is equivalent to ~106,000 MMAE molecules existing within a single cell, sufficient to cause cell death [32, 49]. Nevertheless, the addition of PCMdt-MMAE alters the kinetics of MET/RON internalization in individual cell lines, which could impact the MMAE-mediated cytotoxic activity. Fifth, PCMbs-MR recognizes not only human MET and RON, but also their corresponding homologies in monkeys. This property offers pharmaceutical advantages for using the monkey model to study the PK and toxicological profiles of PCMdt-MMAE.

The PK profile of the mouse provides insights into the dynamics of PCMdt-MMAE in vivo. Our study showed that the PK profile of PCMdt-MMAE fits the two-compartment model with a *t*½ of ~6.3 days, similar to that of other clinically approved ADCs, such as T-DM1 [50, 51]. No differences were observed between the PCMdt-MMAE dynamics in tumor-bearing and tumor-nonbearing mice, indicating that tumor growth does not alter the PCMdt-MMAE PK profile. Moreover, MET and RON overexpression in xenograft tumors does not affect PCMdt-MMAE in vivo. Hence, it can be suggested that tumors constitutively expressing high-to-moderate levels of MET and RON probably have very little impact on the absorption, distribution, metabolism, and excretion of PCMdt-MMAE. Nonetheless, because PCMdt-MMAE does not always recognize the mouse homologs of MET and RON, a PK profile of PCMdt-MMAE in human subjects should be employed to determine whether normal tissues expressing low levels of MET and RON affect the PK profile of PCMdt-MMAE. Regardless of these considerations, PCMdt-MMAE has a favorable PK profile, which provides a pharmaceutical basis for clinical trials to determine its therapeutic efficacy.

An analysis of the toxic effects in Sprague–Dawley rats, an acceptable model for ADC toxicity, indicated that PCMdt-MMAE is relatively safe at therapeutic doses with a minimal impact on animal behavior or bodyweight. However, a single dose of PCMdt-MMAE at 30 mg/kg had a negative impact on animals and causes pathophysiological changes in hematological and blood chemistry indicators. The observed patterns of adverse activities appear to be in line with those observed with MMAE [32, 33]. Toxicological studies analyzing the clinical candidate also indicated that dose-limiting toxicities are related to small molecules, independent of target binding [52, 53]. These toxicities are dose-dependent, moderate, temporary, and reversible. Moreover, a histological analysis of rat tissues did not find any evidence of inflammation, hemorrhage, cell death, structure damage, or weight/size reduction. Considering the average TSC (0.29 mg/kg) of PCMdt-MMAE in the four xenograft models tested, hematological and blood chemistry indicators, and bodyweight changes, a dose of <30 mg/kg should be considered as the dosing guideline for using PCMdt-MMAE in the future.

The biological activities of PCMdt-MMAE in vitro meet therapeutic expectations in terms of its effects on cell cycle, viability, and death (Fig. 4). Three features of PCMdt-MMAE are worth mentioning. First, the effect of PCMdt-MMAE in vitro is target dependent. As cells lacking MET and RON co-expression show minimal death, as evident in Figs. 2d, 4c, and 5d, it can be strongly suggested that the action of PCMdt-MMAE is mediated through MET and/or RON in a target-specific manner. Second, PCMdt-MMAE can effectively inhibit and kill cancer cells expressing MET, RON, and both receptors. PCMdt-MMAE can kill not only cancer cells co-expressing MET and RON, but also those expressing only MET (Hs746T) or RON (SUM52PE) (Fig. 4b, c). Hence, it can be suggested that the MET- and RON-binding arm in PCMbs-MR can interact with MET and RON and induce their internalization for MMAE delivery, leading to cytotoxicity. We speculate that this action can increase the therapeutic scope of PCMdt-MMAE and broaden its targeting activity for cells expressing MET only, RON only, as well as those co-expressing both MET and RON. Third, PCMdt-MMAE is effective in cancer cells exhibiting phenotypic heterogeneity with differential combinations of MET and RON expressions. As shown in tumor samples and cancer cell lines, heterogeneous phenotypes created by differential MET and RON co-expressions with different combinations are extremely complex. The in vitro (Fig. 4) and in vivo (Fig. 5) study results also confirm our hypothesis.

Results obtained using xenograft models prove the efficacy of PCMdt-MMAE in tumors with MET and RON heterogeneous phenotypes. We showed that the therapeutic efficacy of PCMdt-MMAE is comparable to that of PCM-MET01-MMAE and PCM5B14-MMAE. PCMdt-MMAE with a TSC of 0.35 mg/kg reduced the tumor volume by up to 93%, decreased the tumor weight by up to 96%, and eradicated tumors by up to 20% in HT29 tumors expressing high levels of MET and RON (Fig. 5a and Table 2A). In addition, PCMdt-MMAE activity is dose-dependent. In tumors with moderate levels of MET and RON co-expression, 1 mg/kg of PCMdt-MMAE is sufficient to inhibit tumor growth and prevent tumor regrowth for up to two weeks (Fig. 5b, c, and Table 2B). Increasing the PCMdt-MMAE dose to up to 7, 10, and 15 mg/kg resulted in a dramatic inhibition of tumor growth with a superior therapeutic index. Moreover, PCMdt-MMAE inhibited tumor growth mediated by cancer cells from multiple sources, including those from the colon, lung, pancreas, and breast, regardless of their metastatic and chemoresistant status (Fig. 5d). Furthermore, PCMdt-MMAE is effective against tumors with varying combinations of MET and RON expression, which suggests that PCMdt-MMAE exhibits a broad range of anticancer activity, and, hence, it can be used in the treatment of various cancers. We further confirmed the long-lasting effect of PCMdt-MMAE. When administered as a single-dose injection of 10 mg/kg, PCMdt-MMAE blocks tumor growth for almost 4 weeks, which is equivalent to a ~ 6 half-life cycles in vivo (Fig. 5b–d). Thus, PCMdt-MMAE is effective against tumors with a MET/RON heterogeneous expression phenotype.

## Conclusions

Differential expression of MET and RON and their varying combinations in primary PDAC and TNBC is a characteristic feature of cancer heterogeneity. MET and RON dual-targeting ADC PCMdt-MMAE exhibits favorable PK and toxicological profiles and is highly effective against tumors heterogeneously co-expressing MET and RON with their differential combinations. The use of PCMdt-MMAE presents a novel therapeutic strategy to handle cancer heterogeneity.

## Supplementary information

Supplementary Table 1

Supplementary Table 2

Supplementary Table 3

Supplementary Table 4

Supplementary Table 5

Supplementary table legend

## Data Availability

The data supporting the findings of this study are available from the corresponding author upon reasonable request.
