# Supplementary material for: Humanized dual-targeting antibody–drug conjugates specific to MET and RON receptors as a pharmaceutical strategy for the treatment of cancers exhibiting phenotypic heterogeneity
Source: Acta Pharmacol Sin. 2025 Jan 21;46(5):1375–89. doi: 10.1038/s41401-024-01458-7 (PMC12032285; doi:10.1038/s41401-024-01458-7)
Supplement: Supplementary file 1 — Supplementary Table 1 [file 41401_2024_1458_MOESM1_ESM.docx]

| Clinical & Pathological information | Primary PDAC and TNBC samples | |
| --- | --- | --- |
|  | **244 PDAC samples** | **236 TNBC samples** |
| Age (years): mean ± SD | 26-83 (62.97 ± 9.80) | 29-86 （52.81 ± 12.41） |
| Sex |  |  |
| Female | 90 (36.88%) | 236 (100%) |
| Male | 154 (63.11%) | 0 (0.0%) |
| Clinical stage |  |  |
| I | 44 (18.03%) | 125 (52.97%) |
| II | 176 (72.13) | 93 (39.41%) |
| III | 8 (3.28%) | 16 (6.78%) |
| IV | 16 (6.56%) | 2 (0.85%) |
| Tumor Stages |  |  |
| T1 | 4 (1.64%) | 109 (46.19%) |
| T2 | 56 (22.95%) | 77 (32.63%) |
| T3 | 177 (72.54%) | 2 (0.85%) |
| T4 | 7 (2.87%) | 0 (0.0%) |
| Lymph node stage |  |  |
| N0 | 147 (60.25%) | 125 (52.97%) |
| N1 | 97 (39.75%) | 111 (47.03%) |
| Metastatic stage |  |  |
| M0 | 228 (93.44) | 234 (99.15%) |
| M1 | 16 (6.56%) | 2 (0.85%) |
| Cell differentiation |  |  |
| Poor | 18 (7.38%) | N/A |
| Moderate | 168 (68.85%) | N/A |
| Well | 51 (20.90%) | N/A |
| Unknown | 7 (2.87%) | N/A |
| Chemotherapy |  |  |
| No | 120 (49.18%) | 6 (2.54%) |
| Yes | 124 (50.82%) | 230 (97.46%) |

**Supplementary Table 1 Clinical and pathological parameters of primary samples of pancreatic ductal adenocarcinoma and triple negative breast cancer***

*Clinical and pathological information from individual samples was from the Department of Pathology at the First Affiliated Hospital, Zhejiang University School of Medicine. Various tumor parameters were analyzed accordingly as previously described [17, 19].
