# Supplementary material for: Humanized dual-targeting antibody–drug conjugates specific to MET and RON receptors as a pharmaceutical strategy for the treatment of cancers exhibiting phenotypic heterogeneity
Source: Acta Pharmacol Sin. 2025 Jan 21;46(5):1375–89. doi: 10.1038/s41401-024-01458-7 (PMC12032285; doi:10.1038/s41401-024-01458-7)
Supplement: Supplementary file 2 — Supplementary Table 2 [file 41401_2024_1458_MOESM2_ESM.docx]

**Supplementary Table 2 Acute toxicities by a single dose injection**

**of PCMdt-MMAE in Sprague-Dawley rats***

| Sample Measurement | Sprague-Dawley rats | | Duration & levels of ADC toxicity |
| --- | --- | --- | --- |
|  | 10 mg/kg | 30 mg/kg |  |
| Bodyweight/ survival | Normal range/ all survival | <5% decrease/ all survival | Weak effect temporary & reversible |
| Urinalysis | Normal range | Normal range | Not observed |
| Erythrocyte | 8% decrease | ~15% decrease | Weak to moderate effect transient and reversible |
| Leukocyte | ~15% decrease | ~35% decrease | Weak to moderate effect temporary & reversible |
| Neutrophile | ~30% decrease | ~50% decrease | Moderate to strong effect |
| Lymphocyte | ~10% decrease | ~35% decrease | Slight to moderate effect |
| Monocyte | ~5% decrease | ~15% decrease | Slight to moderate effect |
| Alkaline phosphatase | ~1.5-fold increase | ~3.0-fold increase | Slight to moderate effect temporary & reversible |
| Aspartate aminotransferase | ~3.0-fold increase | ~8.5-fold increase | Slight to moderate effect temporary & reversible |
| Alanine aminotransferase | ~2.0-fold increase | ~3.0-fold increase | Slight to moderate effect temporary & reversible |
| Creatine kinase | ~8.5-fold increase | ~40-fold increase | Slight to strong effect temporary & reversible |
| Multiple tissue histological analysis | No abnormalities observed | bone barrow affected | Slight to moderate effect temporary & reversible |

*****PCMdt-MMAE at 10 or 30mg/kg in a single dose was injected into Sprague-Dawley (male, aged at 10 weeks, 3 animals per group). Animals without ADC injection served as the control. All rats were monitored up to 28 days. Blood samples were collected at different time intervals. Hematology and blood chemistry were performed as previously described ^[32]^. The values from control rats were set as the baseline (100%) and used to compare with those from ADC-injected animals.
