# Supplementary material for: Humanized dual-targeting antibody–drug conjugates specific to MET and RON receptors as a pharmaceutical strategy for the treatment of cancers exhibiting phenotypic heterogeneity
Source: Acta Pharmacol Sin. 2025 Jan 21;46(5):1375–89. doi: 10.1038/s41401-024-01458-7 (PMC12032285; doi:10.1038/s41401-024-01458-7)
Supplement: Supplementary file 3 — Supplementary Table 3 [file 41401_2024_1458_MOESM3_ESM.docx]

| Cell line | Tumor Subtype | Histology | Tumor Source | ER/PR/HER2 expression | TP53/BRCA1 status | Other  alterations | Levels of RON expression | Levels of MET expression | Xenograft  Model |
| --- | --- | --- | --- | --- | --- | --- | --- | --- | --- |
| BT-20 | **TNBC (Basal-like-1)** | **IDC** | **Primary breast** | **ER-/PR-/HER2-** | **Wt/Wt** | **Amplified EGFR** | **Negative (0)** | **Moderate (++)** | **ND** |
| DU4475 | **TNBC (IM)** | **DC** | **Pleural effusion** | **ER-/PR-/HER2-** | **Wt/Wt** | **N/A** | **Moderate (++)** | **N/A** | **No** |
| HCC1806 | **TNBC (Basal-like-2)** | **ASCC** | **Primary breast** | **ER-/PR-/HER2-** | **Mut/Wt** | **N/A** | **Negative (0)** | **Negative** | **Yes** |
| HCC1937 | **TNBC (Basal-like-1)** | **IDC** | **Primary breast** | **ER-/PR-/HER2-** | **Mut/Mut** | **PTEN homo deletion** | **High (+++)** | **High (+++)** | **Yes** |
| HCC2185 | **TNBC (LAR)** | **ILC** | **Pleural effusion** | **ER-/PR-/HER2-** | **Wt/Wt** | **PI3KCA mutation** | **High (+++)** | **Negative (0)** | **No** |
| MDA-MB231 | **TNBC (MSL)** | **IDC** | **Pleural effusion** | **ER-/PR-/HER2-** | **Mut/Wt** | **BRAF & KRAS mutations** | **Low (+)** | **High**  **(+++)** | **ND** |
| MDA-MB468 | **TNBC (Basal-like-1)** | **DC** | **Pleural effusion** | **ER-/PR-/HER2-** | **Mut/Wt** | **Amplified EGFR** | **High (+++)** | **Low (+)** | **Yes** |
| SUM52PE | **TNBC (LAR)** | **IDC** | **Pleural effusion** | **ER+/PR-/HER2-** | **Mut/Wt** | **N/A** | **High (+++)** | **Negative (0)** | **Yes** |
| T-47D | **BC** | **IDC** | **Pleural effusion** | **ER+/PR+/HER2-** | **Mut/Wt** | **N/A** | **High (+++)** | **Moderate (++)** | **Yes** |
| Panc-1 | **PDAC** | **DAC** | **Primary pancreas** | **CEA** | **N/A** | **N/A** | **Negative (0)** | **Low (+)** | **Yes** |
| BxPC-3 | **PDAC** | **DAC** | **Primary pancreas** | **CEA** | **N/A** | **N/A** | **Moderate (++)** | **High (+++)** | **Yes** |
| ASPC-1 | **PDAC** | **DAC** | **Metastatic ascites** | **N/A** | **N/A** | **N/A** | **Low-Moderate (+ - ++)** | **High (+++)** | **Yes** |
| FG | **PDAC** | **DAC** | **Metastatic node** | **N/A** | **N/A** | **N/A** | **moderate (++)** | **High (+++)** | **Yes** |
| HT-29 | **CRC** | **AC** | **Primary tumor** | **N/A** | **p53+** | **N/A** | **High (+++)** | **High (+++)** | **Yes** |
| HCT116 | **CRC** | **CC** | **Primary tumor** | **N/A** | **N/A** | **N/A** | **High (+++)** | **Moderate (++)** | **Yes** |
| SW620 | **CRC** | **AC** | **Metastatic node** | **CEA** | **p53+** | **N/A** | **High (+++)** | **Low (+)** | **Yes** |
| Hs746T | **GC** | **AC** | **Metastatic**  **in leg** | **N/A** | **N/A** | **N/A** | **Negative (0)** | **High (+++)** | **Yes** |
| H358 | **NSCLC** | **AC** | **Metastatic alveolus** | **HER2+** | **N/A** | **N/A** | **High**  **(+++)** | **Moderate (++)** | **Yes** |

**Supplementary Table 3 Pathological features of a panel of cancer cell lines used as the model for studying the therapeutic activity of MET-RON dual-targeting ADC PCMdt-MMAE***

*All cancer cell lines were from ATCC. Pathological information such as tumorigenic subtypes, histology appearance, tissue sources, and tumorigenicity in mice were from the documents provided by ATCC. Information about genetic alternations in certain genes and aberrant expression of certain cellular marker proteins were from literatures. Levels of MET and RON expression were from published articles and indicated as negative (0), weak (+), moderate (++), and strong (+++) positivity. AC, adenocarcinoma; ASCC, acantholytic squamous cell carcinoma; BC, breast cancer; BRAC1, breast cancer gene 1; BRAF, raf murine sarcoma viral oncogene homolog B1; CC, carcinoid carcinoma; CEA, carcinoembryonic antigen; CRC, colorectal cancer; DAC, ductal adenocarcinoma; DC, ductal carcinoma; EGFR, epidermal growth factor receptor; ER, estrogen receptor; CRC, colorectal cancer; GC, gastric cancer; HER2, human epidermal growth factor receptor 2; IDC, invasive ductal carcinoma; IM, immunomodulatory; KRAS, Kirsten rat sarcoma viral oncogene homolog; LAR, luminal androgen receptor; MSL, Mesenchymal stem-like; Mut, mutated; NSCLC, non-small cell lung cancer; PDAC, pancreatic ductal adenocarcinoma; PI3KCA, phosphatidylinositol 3-kinase catalytic alpha polypeptide; PR progesterone receptor; PTEN, phosphatase and tensin homolog; TNBC, triple negative breast cancer; TP53, tumor suppressor protein 53; Wt, wild type.
