# Supplementary material for: Humanized dual-targeting antibody–drug conjugates specific to MET and RON receptors as a pharmaceutical strategy for the treatment of cancers exhibiting phenotypic heterogeneity
Source: Acta Pharmacol Sin. 2025 Jan 21;46(5):1375–89. doi: 10.1038/s41401-024-01458-7 (PMC12032285; doi:10.1038/s41401-024-01458-7)
Supplement: Supplementary file 4 — Supplementary Table 4 [file 41401_2024_1458_MOESM4_ESM.docx]

| Cancer cell lines | Changes of cell cycles after PCMdt-MMAE treatment (%) | | | | | | | | | | | | | | | | | |
| --- | --- | --- | --- | --- | --- | --- | --- | --- | --- | --- | --- | --- | --- | --- | --- | --- | --- | --- |
|  | **G0/G1 phase** | | | | | | **S phase** | | | | | | **G2/M phase** | | | | | |
|  | 0h | 6h | 12h | 24h | 36h | 48h | 0h | 6h | 12h | 24h | 36h | 48h | 0h | 6h | 12h | 24h | 36h | 48h |
| **HCC1806** | 63.00 | 61.00 | 60.90 | 64.40 | 63.00 | 61.20 | 16.70 | 17.00 | 14.50 | 12.90 | 16.00 | 17.40 | 20.30 | 22.00 | 24.60 | 22.70 | 21.00 | 21.40 |
| **BXPC3** | 77.00 | 75.20 | 74.70 | 46.50 | 39.10 | 31.20 | 8.80 | 8.30 | 5.40 | 4.60 | 4.10 | 3.70 | 14.20 | 16.50 | 18.70 | 48.90 | 56.80 | 65.10 |

**Supplementary Table 4 Induction of cell cycle changes by PCMdt-MMAE in cancer cells expressing MET and RON***

*Cell cycle analysis was performed as previously described [32]. The percentages of cell cycles in difference phases were calculated from the gated windows as previously described [34].
