# Supplementary material for: Humanized dual-targeting antibody–drug conjugates specific to MET and RON receptors as a pharmaceutical strategy for the treatment of cancers exhibiting phenotypic heterogeneity
Source: Acta Pharmacol Sin. 2025 Jan 21;46(5):1375–89. doi: 10.1038/s41401-024-01458-7 (PMC12032285; doi:10.1038/s41401-024-01458-7)
Supplement: Supplementary file 5 — Supplementary Table 5 [file 41401_2024_1458_MOESM5_ESM.docx]

**Supplementary Table 5 Effect of PCMdt-MMAE on reduction of cell viability in comparisons with anti-MET ADC PCMMET01-MMAE and anti-RON ADC PCM5B14-MMAE in a panel of cancer cell lines***

| Cell lines showing MET & RON | Cancer types | MET expression | RON expression | ADC IC_50_ values for cellular cytotoxicity | | |
| --- | --- | --- | --- | --- | --- | --- |
|  |  |  |  | **Anti-MET PCM-MET01-MMAE** | **Anti-RON PCM 5B14-MMAE** | **Dual targeting PCMdt-MMAE** |
| HCC1806 | TNBC | Negative | Negative | >100 | >100 | >100 |
| HCC1937 | TNBC | ++ | +++ | 1.29 ± 0.25 | 1.51 ± 0.29 | 1.64 ± 0.11 |
| SUM52PE | TNBC | Negative | +++ | >100 | 1.34 ± 0.18 | 1.34 ± 0.29 |
| ASPC1 | PDAC | +++ | + | 0.89 ± 0.16 | 4.24 ± 0.54 | 1.89 ± 0.23 |
| BXPC-3 | PDAC | +++ | ++ | 1.51 ± 0.21 | 3.51 ± 1.01 | 3.45 ± 0.28 |
| FG | PDAC | +++ | +++ | 1.57 ± 0.45 | 2.71 ± 0.32 | 2.18 ± 0.33 |
| Hs746t | GC | +++ | Negative | 0.11 ± 0.05 | >100 | 1.81 ± 0.43 |
| HT29 | CRC | +++ | +++ | 4.91 ± 0.92 | 2.01 ± 0.11 | 2.08 ± 0.07 |
| NIH3T3-RON | Fibroblast | Negative | +++ | >100 | 1.92 ± 0.29 | 3.78 ± 0.38 |
| Average |  |  |  | 1.72 ± 1.51 | 2.46 ± 1.08 | 2.27 ± 0.87 |

*Individual cell lines were treated with different amounts of PCMdt-MMAE for 96h. Cells treated with different amounts of PCM-MET01-MMAE or PCM5B14-MMAE were used for comparison. The MTS assay was used to determine the cell viability. IC_50_ values from individual groups were calculated using GraphPad Prism 6 software as previously described [32].
