# Supplementary material for: Humanized dual-targeting antibody–drug conjugates specific to MET and RON receptors as a pharmaceutical strategy for the treatment of cancers exhibiting phenotypic heterogeneity
Source: Acta Pharmacol Sin. 2025 Jan 21;46(5):1375–89. doi: 10.1038/s41401-024-01458-7 (PMC12032285; doi:10.1038/s41401-024-01458-7)
Supplement: Supplementary file 6 — Supplementary table legend [file 41401_2024_1458_MOESM6_ESM.docx]

**Supplementary Table 1** Clinical and pathological parameters of primary samples of pancreatic ductal adenocarcinoma and triple negative breast cancer.

**Supplementary Table 2** Toxicological studies of PCMdt-MMAE in Sprague–Dawley rats. PCMdt-MMAE, monomethyl auristatin E was conjugated to PCMbs–MR to generate the dual-targeting ADC. ADC, antibody–drug conjugate; PCMdt-MMAE, monomethyl auristatin E was conjugated to PCMbs–MR to generate the dual-targeting ADC.

**Supplementary Table 3** Pathological features of a panel of cancer cell lines used for studying the therapeutic activity of MET-RON dual-targeting ADC PCMdt-MMAE. ADC, antibody–drug conjugate; MET, mesenchymal-epithelial transition; PCMdt-MMAE, monomethyl auristatin E was conjugated to PCMbs–MR to generate the dual-targeting ADC; RON, recepteur d’Origine nantais.

**Supplementary Table 4** Induction of cell cycle changes by PCMdt-MMAE in cancer cells expressing MET and RON. ADC, antibody–drug conjugate; MET, mesenchymal-epithelial transition; PCMdt-MMAE, monomethyl auristatin E was conjugated to PCMbs–MR to generate the dual-targeting ADC; RON, recepteur d’Origine nantais.

**Supplementary Table 5** Effect of PCMdt-MMAE on reduction of cell viability in comparison with anti-MET ADC PCMMET01-MMAE and anti-RON ADC PCM5B14-MMAE in a panel of cancer cell lines. ADC, antibody–drug conjugate; MET, mesenchymal-epithelial transition; PCMdt-MMAE, monomethyl auristatin E was conjugated to PCMbs–MR to generate the dual-targeting ADC; RON, recepteur d’Origine nantais.
